# The effects of heatwaves on hospital admissions in the Edirne province of Turkey: A cohort study

**DOI:** 10.1097/MD.0000000000034299

**Published:** 2023-07-14

**Authors:** Yunus Ozturk, Hakki Baltaci, Bulent Oktay Akkoyunlu

**Affiliations:** a Marmara University, Institute of Pure and Applied Sciences, Occupational Safety, Istanbul, Turkey; b Gebze Technical University, Institute of Earth and Marine Sciences, Gebze, Kocaeli, Turkey; c Marmara University, Department of Physics, Istanbul, Turkey.

**Keywords:** cardiology, heat waves, hospital admissions, policlinic

## Abstract

Studies show that heat waves (HWs) are among the most important atmospheric phenomena that negatively affect human health. This study aims to determine the effects of HWs on hospital admissions (HA) in the Edirne province of Turkey. Polyclinic admission and atmospheric data, including daily maximum temperatures, were used. HW is defined as temperature at the % 90 threshold of daily maximum temperatures that persists for at least 3 consecutive days or more. With this definition, a 6-day HW was detected, and a lag of 3 days was added to this HW. Logarithmic Z test was used for the analysis. As a result of the study, The Risk Ratio (RR) showing the relationship between 9-day HW and HAs was calculated as 1.19 (95% confident interval [CI]: 1.17–1.21, *P* < .05), and it was determined that there were 2557 extra HAs in total. When HAs were analyzed according to sex, it was observed that female admissions were higher than male admissions. To analyze admissions by age, the data were divided into 3 groups: children (<15 years), adult (15–64 years), and elderly (≥65 years). As a result of the analysis, the highest increase was observed in patients < 15 years of age, and the RR was 1.33 (95% CI: 1.24–1.42 P < .05). When the patient density in polyclinics was analyzed, the Cardiology polyclinic had the highest number of patient admissions with an RR, 1.36 (95% CI: 1.30–1.43 *P* < .05). The results of this study will guide measures to be taken against HWs.

## 1. Introduction

In the 21^st^ century, climate change is projected to increase both mean and extreme temperatures. As a result, heat waves (HW) as a natural disaster will become more frequent, intense and long-lived. With the increasing frequency and severity of HWs, significant increases are expected in the mortality and morbidity rates, and applications to the emergency services of the hospitals. Under climate change, new and emerging risk factors together with heat stress conditions were studied in detail.^[1–3]^ With this global health threat in this century, for example, COVID-19 pandemic, comprehensive strategies and new challenges should be developed.^[4,5]^ From changing extreme atmospheric conditions under global climate change, HWs and their influence on human health is one of the important issues to be understandable. For this reason, many studies have investigated the effects of HWs on human health in recent years.^[6–9]^ These studies determined that there was an increase in deaths,^[10–13]^ hospital and emergency room admissions^[14–17]^ and ambulance calls^[18,19]^ during the HW period. In these studies, women,^[20–22]^ the elderly^[23–25]^ and children^[26]^ were the most affected group by HWs. In addition, HW has been found to affect people with chronic diseases such as heart,^[27,28]^ kidney failure,^[29]^ and respiratory failure.^[30]^

Studies investigating the HW-health relationship have also been conducted in Turkey. A study conducted in Istanbul found that 419 additional deaths occurred in the HWs between 2013 and 2017.^[27]^ In 2016, 29 additional deaths occurred in HWs in Izmir.^[31]^

Although these 2 studies were conducted in Turkey, they were not considered as sufficient to demonstrate the effects of HWs on human health. Recent studies have shown an increase in the number and duration of HWs in Turkey.^[32]^ Therefore, further studies based on the HW-health relationship should be investigated in detail.

This study aims to reveal the effects of HWs on human health in Edirne city, which is located in Northwest of Turkey. Therefore, changes in the number of patients in hospital outpatient clinics during the HWs period were examined. The study was conducted to determine patients’ exposure to high temperatures based on their age, sex, and chronic diseases. The results obtained from this study will be helpful for future studies on protection against HWs.

## 2. Material and Method

### 2.1. Study area

Edirne is located between 40° 30’ and 42° 00’ N latitude and 26° 00’ and 27° 00’ E longitude in the Thrace section of the Marmara Region. It is 41 m above sea level and has a surface area of 6.279 km^2^, with a population of 408,000 people. It has a Mediterranean climate, with hot and dry summers and mild and rainy winters. The hottest month in the region is July, with an average temperature of 44 centigrade celsius (°C). The coldest month is January, with an average of −19.5°C. The average annual temperature is 14°C.

### 2.2. Hospital data

Hospital data were obtained with permission from Edirne Provincial Health Directorate. These data included the number of daily polyclinic admissions, sex, age, and patient diagnoses in all hospitals in Edirne, 2018. As hospital polyclinics in Turkey are closed during public holidays, no weekend data are available.

### 2.3. Meteorology data

Meteorological data used for the analysis were obtained from the Turkish State Meteorological Service. Daily maximum temperature records from the Edirne meteorology station between 1960 and 2019 were used for the analysis. The study interval was from May 1 to September 30, when high temperatures were recorded. Instead of classic summer months period (June-August), we used extended summer months, (May-September) because, temperature records began to be broken frequently in this 5-month period.

### 2.4. HW definition

Since no single definition of HW exists, many definitions have been used in previous studies.^[33]^ In these definitions, daily maximum,^[34,35]^ minimum,^[36]^ and average temperatures^[37–39]^; relative values such as 90% and 95%^[21–40]^ or constant thresholds such as 28°C and 35°C,^[41]^ and consecutive temperatures such as 2 days, 3 days or 4 days^[42,43]^ were used. Our study defined HWs as temperatures that persist for 3 consecutive days or more at 90% as a threshold value using daily maximum temperatures. According to this definition, in 2018, HW, which lasted for 6 days between August 30 and September 4, was detected in Edirne. In previous studies, lag days were added to HW events when the effects of high temperatures were observed.^[27]^ For this reason, 3-lag days were added to the 6-day HWs, and a total of 9 HWs days were used in the analysis.

### 2.5. Statistical analysis

Microsoft Office Excel (Microsoft Corporation, Redmond, WA) and R statistical program was used for statistical analyses. Increases in hospital admissions (HA) during 3-lag day HWs periods were calculated using Equations 1 and 2. Risk Ratios (RRs) and their significance were calculated using Equation 3 and 95% confidence intervals were also calculated. Statistical significance was set at *P* < .05. Natural logarithmic Z tests were used to compare HA rates in the 3-day lagged HW period with those in the reference periods.^[27]^ The Z Test was calculated using Equation 6, and the *P*-value was calculated using Equation 7.

$$HA_{\left(HeatWave\right)}=\;\frac{Number\;of\;The\;Hosp.\;\;Ad.\;\;Heatwave\;Period}{Population*Number\;of\;Days\;Heatwave\;Periods}$$ (1)

$$HA_{\left(Referance\;Period\right)}=\;\frac{Number\;of\;The\;Hosp.\;\;Ad.\;\;Referance\;Reriod}{Population*Number\;of\;Days\;Referance\;Periods}$$ (2)

$$RR=\frac{HA_{\left(Heatwave\right)}}{HA_{\left(Referance\;Period\right)}}$$ (3)

$$var\left(HA_{Heatwave}\right)=\sqrt{\frac{Numbers\;of\;Hosp.\;\;Ad.\;\;Heatwave\;Period}{{\left(Population*Number\;of\;Days\;Heatwave\;Periods\right)}^{2}}}$$ (4)

$$var\left(HA_{Referance\;Period}\right)=\sqrt{\frac{Numbers\;of\;Hosp.\;\;Ad.\;\;referance\;Period}{{\left(Population*Number\;of\;Days\;Referance\;Periods\right)}^{2}}}$$ (5)

$$Z=\frac{\ln\left(HA_{heat\;wave}\right)-\text{ln}\left(HA_{referance\;period}\right)}{\sqrt{\frac{var\left(HA_{heat\;wave}\;\right)}{{\left(HA_{heat\;wave}\right)}^{2}}\;\;+\;\;\frac{var\left(HA_{referance\;period}\right)}{{\left(HA_{referance\;period}\right)}^{2}}}}$$ (6)

P Value=2x(1−Normdist(Z,avarage; standard deviation;cumulative) (7)

Here, HA_(Heatwave period)_ indicates the HA rate during the HW periods that occurred in 2018, HA (_Reference period)_ indicates the HA rates during the reference periods. The analyses compared hospital data recorded during the 9-day HW between August 30 and September 7, 2018, with hospital data recorded during the reference periods. Hospital data from August 6 to 29 before the HW and hospital data from September 10-October to 3 after the HW were used as references for the analysis.

## 3. Results

Table 1 shows the statistical information on the temperatures and hospital data for the Edirne. Accordingly, the highest daily average temperature was 25.5°C in August. The highest daily average number of patient applications in polyclinics was recorded in August. Cardiology polyclinic applications were highest in September, whereas pulmonology and neurology polyclinic applications were highest in August. The highest number of female patient applications were received in August, whereas the highest number of male patient applications were received in September. Most patients in the 0 to 14 age group were admitted to polyclinics in September, while most patients in other age groups were admitted in August.

**Table 1:** Descriptive statistics by yr.

	July 2018	August 2018	September 2018
Daily average temperatures (°C)	25.3 (18.2–32.7)	25.5 (18.3–33.1)	20.6 (14.2–27.9)
Daily average number of polyclinics patients admissions	2306	2593	2519
Average number of patient admission in cardiology polyclinic	273	263	300
Average number of patient admission in chest diseases polyclinic	273	334	316
Average number of patient admission in neurology polyclinic	282	337	327
Average number of patient admission of other polyclinics	1479	1659	1575
The daily average number of patients to polyclinics admissions by gender			
Women	1251	1446	1363
Men	1055	1147	1156
The daily average number of patients to polyclinics admissions by age			
<15	127	153	157
15–64	1557	1751	1702
>=65	623	688	647

°C = centigrade celsius.

### 3.1. HW and HA

The RR showing the association between the 9-day HW that occurred between August 30 and September 7, 2018, and all polyclinic admissions in the hospital was calculated as 1.19 (95% confident interval [CI]:1.17–1.21, *P* < .05). Table 2 shows the RRs and confidence intervals for the increase in hospital polyclinic admissions of HW-affected patients by age and sex. Chronic diseases most affected by HWs include heart, respiratory, high blood pressure, and kidney diseases.^[44]^ Accordingly, the RRs showing the relationship between HW in 2018 and the number of patient applications by polyclinics were determined as follows: It is 1.17 (95% CI:1.11–1.23, *P* < .05) in the pulmonology polyclinic, 1.36 (95% CI:1.30–1.43, *P* < .05) in the cardiology polyclinic and 1.24 (95% CI:1.18–1.30, *P* < .05) in the neurology polyclinic.

**Table 2:** Hospital admissions during 2018 heat waves periods.

		Number of hospital applications in the HWs period	Average of hospital admissions in the reference period	RR	Confidents intervals (%95 CI)		*P* value
All polyclinics		16376	13819.0	1.19	1.17	1.21	.0000
Chest diseases polyclinic		2019	1728.2	1.17	1.11	1.23	.0000
Cardiology polyclinic		2081	1526	1.36	1.30	1.43	.0000
Neurology polyclinic		2201	1778	1.24	1.18	1.30	.0000
By gender	Women	8976	7476	1.20	1.17	1.23	.0000
	Men	7400	6357	1.16	1.13	1.19	.0000
By age	<=14	1088	820	1.33	1.24	1.42	.0000
	15–64	10907	9404	1.16	1.14	1.18	.0000
	>=65	4381	3595	1.22	1.18	1.26	.0000

RR = risk ratio.

**Figure 1.**
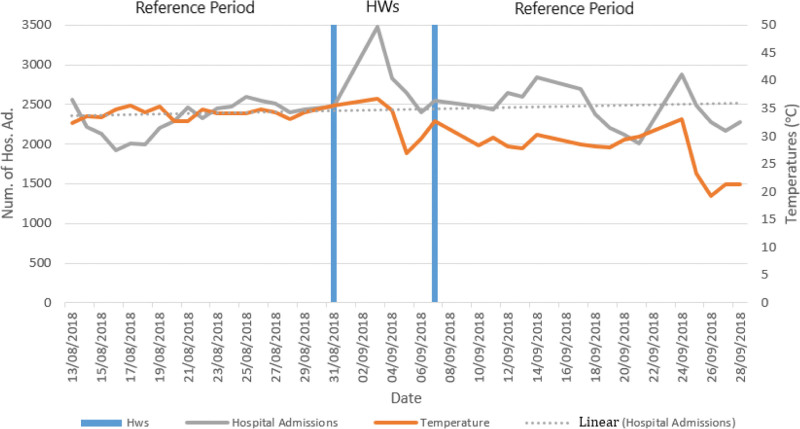
Heatwave and hospital admissions in Edirne province in 2018.

Outpatient visits during the HW period were also analyzed by sex. Accordingly, the RRs for female and male patient applications during the HW period were calculated as 1.20 (95% CI:1.17–1.23 *P* < .05) and 1.16 (95% CI:1.13–1.19 *P* < .05), respectively. When polyclinic visits were analyzed by age, the RRs were calculated as 1.33 (95% CI:1.24–1.42 *P* < .05) for the children, 1.16 (95% CI:1.14–1.18 *P* < .05) for the adult and 1.22 (95% CI:1.18–1.26 *P* < .05) for the elderly. Figure 1 shows the relationship between 9-day HW and HAs in 2018.

The RRs and confidence intervals showing increases in the number of chronic patients recorded in hospital polyclinics during the HW are shown in Table 3. Accordingly, the disease with the highest increase during the HW period in the cardiology polyclinic was I25, with a RR of 1.77 (95% CI:1.44–2.18 *P* < .05). The other diseases most affected by HW were E78, with a RR of 1.56 (95% CI:1.29–1.89 *P* < .05), and I49, with a RR of 1.47 (95% CI:1.3–1.66, *P* < .05).

**Table 3:** Polyclinics admissions during 2018 heat waves periods.

		ICD CODE	Number of polyclinic admissions during HWs period	Average of polyclinics admissions during the reference period	RR	Confidents intervals (%95 CI)		*P* value
Cardiology polyclinic	Atherosclerotic cardiac disease	I25	120	58	1.77	1.44	2.18	.000
	Atrial fibrillation and flutter	I48	260	240	1.16	1.02	1.33	.029
	Essential (primary) hypertension	I10	510	410.6	1.24	1.13	1.37	.000
	Chest pain	R07	110	76	1.44	1.17	1.78	.001
	Hyperlipidemia	E78	136	87	1.56	1.29	1.89	.000
	cardiac arrhythmia	I49	340	231	1.47	1.3	1.66	.000
Chest diseases polyclinic	Asthma	J45	657	569.8	1.15	1.06	1.26	.001
	Chronic obstructive pulmonary disease (COPD)	J44	364	279	1.30	1.16	1.46	.000
	Bronchitis	J20	244	224	1.09	0.95	1.25	.226
	Pneumonia	J18	102	117	0.87	0.71	1.08	.207
	Allergic Rhinitis	J30	426	201	2.12	1.89	2.37	.000
Neurology polyclinic	Cerebrovascular disease	G46	300	227.6	1.32	1.16	1.5	.000
	Headache syndromes	G44	283	228	1.24	1.09	1.41	.001
	Disorders of the central nervous system	G96	146	92.4	1.58	1.31	1.9	.000
	Polyneuropathies	G62	108	86	1.25	1.01	1.55	.036
	Generalized anxiety disorder	F41	138	114	1.21	1	1.46	.046

RR = risk ratio.

In the pulmonology polyclinic, the 3 diseases with the highest increase during the HW period were J30 with a RR of 2.12 (95% CI:1.89–2.37, *P* < .05), J44 with a RR of 1.30 (95% CI:1.16–1.46 *P* < .05), and J45 with a RR of 1.15 (95% CI:1.06–1.26, *P* < .05). In the neurology diseases polyclinic, the most frequently recorded diseases and RRs were G96 1.58 (95% CI:1.31–1.9 *P* < .05), G46 1.32 (95% CI:1.16–1.5 *P* < .05), and G62 1.25 (95% CI:1.01–1.55 *P* < .05), respectively.

## 4. Discussion

In our study, hospital data admissions for Edirne province were used from the 2018 year. The limitation of the study is that we work with the relatively few HA data and the absence of records on holidays and weekends. Besides, we used statistical analysis for the patients who were admitted to the hospital. Therefore, we do not have information about the patients who were not admitted to the hospital. Apart from this, it is very important in terms of showing the continental climate feature of the studied area, being significantly affected by HWs, and the economic income mainly depends on agricultural activities (workers in open areas are more exposed to HWs), For this reason, it is very important to reveal the relationship between the health diseases and HWs to raise awareness among the public and public authorities. According to our results, 6 HW days were recorded between August 30 and September 4, 2018. Adding 3-lag days to this HW increased its duration to 9 days. During this period, it was found that there was a 19% increase in total HAs, and a total of 2557 extra admissions occurred. These results are consistent with those of previous studies. In a study conducted in Izmir, Turkey, a 19% increase in emergency department admissions during HW periods was reported.^[31]^ A study conducted in Brisbane, Australia reported a 14% increase in emergency department visits during the HW period.^[33]^ A study conducted in Sydney, Australia reported a 2% increase in emergency room admissions during HW periods.^[17]^ In August 2003, a study was conducted in France to investigate the increase in the number of HAs to emergency departments and deaths during HW. This study observed 2600 more Emergency Department visits and 1900 more HAs.^[45]^ In 2006, HW occurred in California, the USA. Hospitalization and emergency department records were collected from 6 regions in California.^[46]^ During HW, 16166 extra emergency room visits and 1182 extra hospitalizations were detected. In 2011, a HW occurred in New Wales, Australia, lasting approximately 1 week. This study showed a 14% increase in ambulance calls and a 2% increase in emergency department admissions.^[18]^ During an HW in Shanghai, China, in 2013, emergency room visits and ambulance calls increased by 2.65% and 4.85%, respectively.^[19]^

Looking at HAs by sex during the HW period, it was determined that there was a higher increase in admissions of female patients than in male patients. In this regard, it was determined that there was a 20% increase in the admissions of female patients during the HW period. This result is consistent with those of previous studies. A study on the impact of HWs on health in 9 European cities found that women were more affected group by high temperatures.^[21]^ In a study conducted in 7 cities in Korea, it was determined that women were more affected by HW than men.^[22]^ For Turkey, it was determined that the proportion of female patients was higher than male patients in the emergency service applications during HW periods.^[31]^ A study conducted in Barcelona found that elderly female were more affected by high temperatures during the HW period.^[20]^ A study of 9 European cities examining the relationship between HW and health found that women aged 75 years and older were more affected by high temperatures than were men.^[21]^ In 3 European cities, women aged ≥65 years were the most affected by HWs.^[23]^

HAs during the HW period were analyzed according to age. Therefore, the hospital data were divided into 3 groups: children, adult, and elderly. The analysis revealed that patients aged <15 and ≥65 years were the most affected group during the HW period. Accordingly, the increases in HAs of patients aged ≤15 and ≥65 years in the 4 HW periods were 33% and 22%, respectively. These results are consistent with those of previous studies. A study conducted in the UK found that the elderly and children were more affected by HWs.^[26]^ A study conducted in Australia found a 13% increase in emergency department admissions in the 0 to 15 age group during HW.^[47]^ A study conducted in Italy found that patients aged ≥75 years were more vulnerable to HW.^[24]^ In 2003, the effects of HW in Europe were analyzed by gender and age.^[48]^ Accordingly, it was found that female patients aged ≥65 years were more severely affected by high temperature. A study conducted in Australia found that patients aged ≥65 years were more likely to be affected by HW.^[49]^ A study conducted in Italy found a 13% increase in HAs of patients aged ≥75 years during HW periods.^[16]^ A study conducted in Portugal determined that the age group most affected by high temperatures is 65 years and older.^[25]^ Research conducted in Belgrade concluded that patients aged >75 years were the most affected by HWs.^[50]^

Considering polyclinics with respect to patient applications, the polyclinic with the highest number of applications was cardiology. There was A 36% increase in patient admissions to the cardiology polyclinic. In this polyclinic, the increases in the 4 diseases with the highest increase during the HW period were 77% for atherosclerotic heart disease, 56% for hyperlipidemia, 47% for cardiac arrhythmia, and 44% for chest pain. These results are consistent with those of previous studies. A study conducted in the USA determined that the majority of deaths during the HW period were due to heart disease.^[51]^ A study conducted in China found that deaths due to heart disease increased during the HW period.^[52]^

In neurology polyclinics, HW increased by 24%. The disease increases recorded in this outpatient clinic during the HW period were 58% for central nervous system disorders, 32% for cerebrovascular diseases, 25% for polyneuropathies, and 24% for headaches. These results coincide with those of a study conducted in Beijing, China, which found a 93% increase in the deaths of patients with cerebrovascular disease during the HW period.^[53]^ A study conducted in Spain determined that one of the causes of infant mortality during HW periods was nervous system disorder.^[54]^

There was a 17% increase in pulmonology polyps. The most common diseases recorded in this outpatient clinic during the HW period were Allergic Rhinitis, Asthma, Chronic obstructive pulmonary disease (COPD), etc, respectively. During the HW period, the incidences of allergic rhinitis, COPD, and asthma increased by 112 %, 30%, and 15%, respectively. These results are consistent with those of previous studies. One study determined that increases in respiratory and allergic diseases occurred because of the deterioration in air quality during the HW period.^[55]^ A study in Beijing found a 93% increase in admissions of patients with respiratory problems during HW periods.^[30]^ Another study found an increase in diseases, such as asthma, rhinosinusitis, COPD, and respiratory infections, due to climate change.^[56]^

## 5. Conclusion

This study showed an association between HWs and HAs in northwestern Turkey. During the HW period, there was a 19% increase in total HAs, and 2557 extra admissions were reported. When the applicants were analyzed by sex, it was found that there was a 20% increase in the number of female patients compared with male patients. Regarding the age of the applicants, it was determined that the children were the most affected group by HWs, and there was a 33% increase in this age group during the HW period. There was a 22% increase in the number of applications for elderly patients. Considering the increase in polyclinics, the highest increase was observed in the cardiology polyclinics, with an increase of 36%. In this polyclinic, there was a 77% increase in the number of patients with atherosclerotic heart disease.

## Acknowledgments

We would like to thank the Marmara University Scientific Research Projects Unit for the “FDK-2021-10257” project support number.

## Author contributions

**Writing – original draft:** Yunus Ozturk.

**Writing – review & editing:** Hakki Baltaci, Bulent Oktay Akkoyunlu.
